# Mechanism of the Curative Effect of Wen-Shen-Jian-Pi Prescription in the Treatment of Amyotrophic Lateral Sclerosis

**DOI:** 10.3389/fnagi.2022.873224

**Published:** 2022-04-08

**Authors:** Fan Gong, Wei Zhu, Weilong Liao, Mingzhe Wang, Xuanlu Zheng, Chenghui Wang, Te Liu, Weidong Pan

**Affiliations:** ^1^Department of Neurology, Shuguang Hospital Affiliated to Shanghai University of Traditional Chinese Medicine, Shanghai, China; ^2^Department of Neurology, Gongli Hospital Affiliated to Naval Medical University, Shanghai, China; ^3^Neurology Department of Integrated Chinese and Western Medicine, Shanghai Pudong TCM Hospital, Shanghai, China; ^4^Shanghai Geriatric Institute of Chinese Medicine, Shanghai University of Traditional Chinese Medicine, Shanghai, China

**Keywords:** amyotrophic lateral sclerosis, Wen-Shen-Jian-Pi prescription, glutamate receptor, AR2 mouse, mitochondria

## Abstract

**Objective:**

To study the mechanism of the effect of Wen-Shen-Jian-Pi (WSJP) prescription on an ALS model comprising mice knocked out for an encoding RNA editing, mice (AR2).

**Methods:**

Twenty-four transgenic AR2 mice were randomly divided into a vehicle group, a low dose WSJP group (15 mg), a medium-dose WSJP group (30 mg), and a high-dose WSJP group (45 mg) (all *n* = 6 per group). In the treatment groups, the WSJP prescription was given once a day while the vehicle group was fed the same volume of water. The weekly changes in body weight, rotarod test, and grip strength were used to detect the changes in the AR2 and changes of the number of normal mitochondria, abnormal mitochondria, and autophagosomes in injured spinal cord cells were used to evaluate the pathogenetic effects of WSJP treatment.

**Results:**

The WSJP-treated AR2 mice gained weight more quickly from 8 weeks, and showed active behavior and displayed significantly better constant rotarod scores and grip strengths during the experiment compared with those of the vehicle AR2 mice. The number of normal mitochondria in the WSJP-treated AR2 mice had significantly more normal mitochondria than the vehicle group, while the numbers of abnormal mitochondria and autophagosomes were greatly decreased compared with those in the vehicle group.

**Conclusion:**

The WSJP prescription could delay the decline in motor function of ALS model mice by reducing the degeneration of neurons. The potential of WSJP to treat ALS should be assessed in a clinical trial.

## Introduction

Amyotrophic lateral sclerosis (ALS) is the most frequently diagnosed adult-onset motor neuron disease. ALS involves the progressive loss of upper and lower motor neurons. Within a few years of developing ALS, patients die from progressive respiratory muscle paralysis; unfortunately there are no currently available therapies that can alter the disease course effectively (Duffy et al., 2011; Brown and Al-Chalabi, 2017; Aizawa et al., 2021). Whether it is sporadic ALS or familial ALS, because of its involvement of progressive muscle weakness, and even atrophy, in Traditional Chinese medicine (TCM), ALS should be classified as an “amyotrophy and weakness disease” (Gao et al., 2017). According to its pathogenesis, TCM doctors think that the disease is mainly caused by deficiency of some related organs, which deficiencies are mainly concerned with the yang of the spleen and kidney, mixed with deficiency of the stomach, liver, and lung, and weakness or deficiency of qi (one the circulating energy materials in TCM theory). Therefore, treatment methods for ALS involving tonifying the yang of the kidney and spleen, and invigorating qi in China suggested a certain clinical efficacy (Wang et al., 2022). According to the theory of TCM differentiation and TCM constitution theory, we followed-up of nearly 500 patients with ALS and found that in the early stage of ALS, deficiency the yang of the spleen and kidney is the main syndrome type, together with deficiency of the stomach, liver, and lung; and in the middle and late stage, in addition to deficiency the yang of the spleen and kidney, syndromes of deficiency and excess are mostly intermingled, accompanied by blood stasis and phlegm (Grimm and Eckert, 2017). According to the classic text of traditional Chinese medicine *Huang Di Nei Jing*, the “spleen controls the main muscle function of limbs,” “qi is flowing energy” and the “kidney fills and manages circulation of energy to the muscle as the officer” (Hosaka et al., 2019). The long-term clinical experience of the Shanghai University of TCM led to the use of pure TCM herbs, named the Wen-Shen-Jian-Pi (WSJP) prescription, to delay the progression of ALS and improve the clinical symptoms of patient with ALS (Kwak et al., 2010; Jin et al., 2019); however, the mechanism of action of WSJP is not clear.

A study of ALS indicated that the toxicity mechanism is mediated by GluR2 [Glutamate Ionotropic Receptor α -amino-3-hydroxy-5-methyl-4-isooxazole-propionic acid (AMPA) Type Subunit 2], which plays an important role in both the pathogenesis of sporadic ALS and animal models of superoxide dismutase 1 (SOD1)-related familial ALS (Liu et al., 2018). Based on research by Professor Shin, we successfully made an ALS model comprising mice knocked out for *Adar2* (encoding the RNA editing, enzyme adenosine deaminase acting on RNA 2), referred to as AR2 mice, which can reflect the occurrence and progression of ALS (Mehta et al., 2021). The AR2 model was treated with the WSJP prescription to observe its effect on the motor function of AR2 mice, and the mechanism of action of the observed effect was investigated.

## Materials and Methods

### Animals

The conditional homozygous *Adar2* knockout mice (*ADAR2flox/flox/Vacht*-Cre.Fast; AR2) were used in this study (Mejzini et al., 2019). Intercrosses of *Adar2 flox/* + */Vacht*–Cre. Fast mice produced *Adar2 flox/flox*/*Vacht*–Cre.Fast (AR2) mice, which are either heterozygous or homozygous for the Cre transgene, which uses the vesicular acetylcholine transporter gene (*Vacht*) promoter to direct restricted Cre expression in cholinergic neuron subsets, including spinal motor neurons. Heterozygous or homozygous *Vacht*–Cre mice had similar Cre expression levels. This study used both male and female AR2 mice, in which selective expression of Cre in motor neurons is under the control of the *Vacht* promoter, which ablates the *Adar2*flox gene in approximately half of motor neurons at five postnatal weeks. DNA was obtained from Tail biopsies and used for PCR-based genotyping. Consequently, at the Q/R site in the motor neurons of AR2 mice, 100% and no more than 30% of *Glua2* genes were unedited (Pan et al., 2013a,Pan et al., 2021). In the AR2 mice, *Glua2* expression from an unedited Q/R site causes the slow progressive death of motor neurons by mechanisms related to the Ca_2+_-permeable AMPA receptor.

Two to three mice were housed in each cage and subjected to a 12:12 h light-dark cycle with food and water *ad libitum* (Pan et al., 2011). The animal experiments were carried out following the tenets of the Declaration of Helsinki, the Guidelines of Animal Studies of the Shanghai University of Traditional Chinese Medicine, and the National Institutes of Health of China. The Committee of Animal Handling of the Shanghai University of Traditional Chinese Medicine also approved the experimental procedures.

### Treatment With the Wen-Shen-Jian-Pi Prescription

The Wen-Shen-Jian-Pi prescription (WSJP) was provided by the pharmaceutical laboratory of Shuguang Hospital, and consisted of seven traditional Chinese herbs *(Radix Jinseng*, 15 g; *Radix Astragali*, 15 g; *Radix herba Cistanchedeserticola*, 30 g; *Rhizama Atractylodis Macrocephala*, 15 g; *Poria Cocos*, 15 g; *Radix Glycyrrhiza*, 10 g; and *Herba Epimedii*, 25 g). The materials (Batch number JL-SC-031-01) were prepared by Xu Chongdao Chinese Herbal Pieces Factory of Shanghai Yaofang Co., Ltd., (Shanghai, China) according to the Good Manufacturing Practice production procedure (SMP-SC-012, 2010, China). The materials were decocted for 1 h using 500 mL of distilled water, the supernatant was filtered, added to 500 mL of distilled water, and incubated for 1 h. The solution was concentrated to 50 mL using gentle heat, cooled, added with anhydrous ethanol, stirred using a magnetic stirrer, and centrifuged at 25,000 × *g* for 20 min. The supernatant was retained, the ethanol was evaporated at a constant temperature, and the decoction was made up to a concentration of 1 g/mL with distilled water. After autoclaving, the decoction was stored in refrigerator at −20°C for later use. The main components of the decoction were analyzed and identified using high performance liquid chromatography. The administration dose refers to the conversion formula for human to mouse doses: mouse dose = human dose × 0.71/0.11. Mice received the decoction daily *via* oral gavage. Before the 14-day test, AR2 mice at 17 weeks old received the WSJP prescription at 15, 30, and 45 mg/kg (*n* = 5 per concentration), respectively, for 12 weeks. Before the 14-day test, the control AR2 mice (*n* = 5) received the same volume of water for 12 weeks *via* oral gavage.

### Transmission Electron Microscopy

Spinal cord cells were evaluated histologically using TEM. Mouse tissues were fixed and embedded following previously described methods (Pan et al., 2013b). Then, 70 nm thick slices were obtained and were attached to copper grids, stained with 1% uranium acetate and 1% lead citrate (both Sigma-Aldrich, St. Louis, MO, United States), and then observed using a JEM-1230 transmission electron microscope (JEOL Ltd., Tokyo, Japan).

### Performance Analyses

All the performance experiments were completed on three consecutive working days. The first 2 days were the training period, and the third day was the formal test day. The mice were transferred from the feeding room to the testing site 30 min before the test for adaptation to the environment. The measurements were repeated three times for each mouse.

#### Rotarod Task

The animals were placed on a rotarod (Shanghai Yi Shu Information Technology Co., Ltd., RD1123-RS-M, China). The longest test time was 300 s. In a test, if the mice reached this time without falling off in a test, there was no need to repeat the test and the experiment was complete. At the end of the experiment, the data obtained were analyzed.

#### Grip Strength

A researcher held the mouse by its tail and allowed it to grab the steel grip of the baseplate of the Grip Strength Meter using its forepaws (Chengdu Taimeng Software Co., Ltd., FT-2000, China). The mouse was then pulled back gently until it released the steel grip. We recorded the average power (N) at the time of grip release of three trials.

A researcher who was blinded to the drug administration information carried out the behavioral measurements weekly.

### Statistical Analyses

Data are shown as the mean ± the standard error of the mean (SEM). SPSS 19.0 software (IBM Corp., Armonk, NY, United States) was used to carry out the statistical analyses. For comparisons between groups, the Wilcoxon rank sum test was employed. For comparisons among multiple groups, one-way analysis of variance (ANOVA) followed by Tukey *post hoc* tests were used. A *p* value less than or equal to 0.05 was considered as statistically significant.

## Results

### Wen-Shen-Jian-Pi Prescription Prevents Weight Loss in Amyotrophic Lateral Sclerosis Model Mice

**FIGURE 1:**
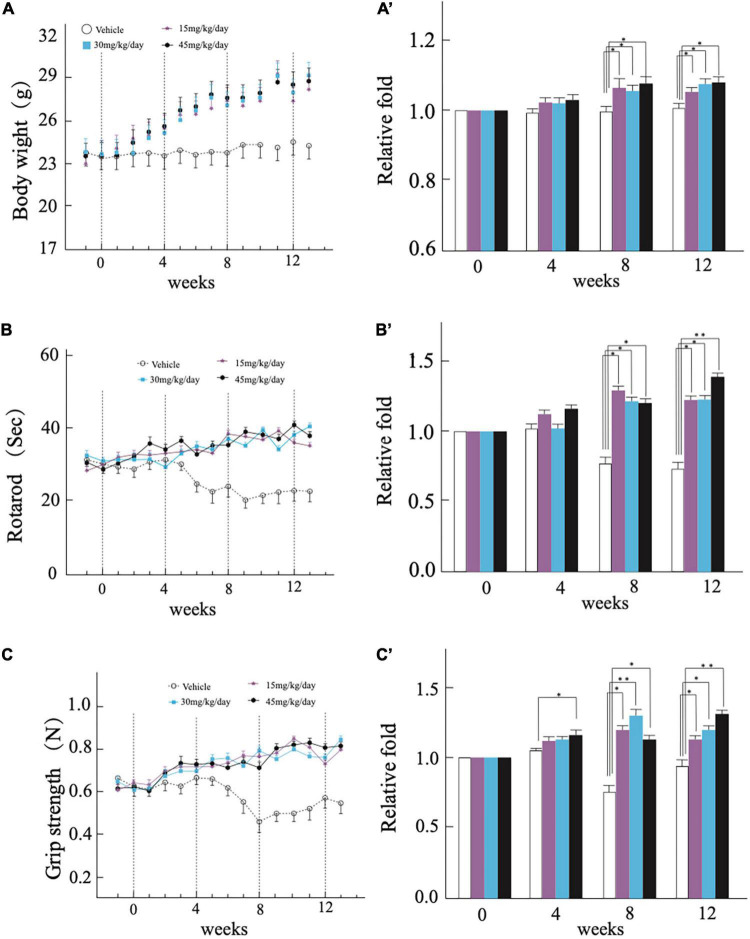
Continuous treatment with Wen-Shen-Jian-Pi (WSJP) prescription for 12 weeks to rescue motor dysfunction in AR2 mice. Changes in **(A)** mouse body weight, **(B)** latency of the mouse to fall on the rotarod task, **(C)** mouse grip strength each week during the experiment for the WSJP-treated AR2 mice (*n* = 18; *n* = 5 male, *n* = 13 female) and vehicle-treated AR2 mice (*n* = 6; *n* = 2 male, *n* = 4 female). Panels **(A′–C′)** indicate the relative fold change for each group. Data are shown as the mean ± the standard error of the mean (SEM). *p < 0.05, **p < 0.001 compared with time zero at each subsequent time point.

We administered the WSJP prescription to 17-weeks-old homozygous (AR2) conditional *Adar2* knockout mice orally each day for the whole administration period. Mice receiving the same volume of water (vehicle) were used as controls. At the beginning of the experiment, among the groups of mice, there were no significant differences between the time of mice. With time, all of the mice gradually gained body weight. Compared with the vehicle group, the WSJP-treated AR2 mice gained weight faster. Before 8 weeks, the difference in the weight gain between the WSJP-treated AR2 mice and the control AR2 mice was not significant, but was significant after 8 weeks. There were no significant differences in weight gain among the three WSJP-treated groups over the whole experimental period (Figures 1A,A′).

### Wen-Shen-Jian-Pi Prescription Improves Behavioral Functions in Amyotrophic Lateral Sclerosis Model Mice

The mice were subjected to rotarod retention time and grip strength tests each week during the experimental period. The WSJP-treated AR2 mice were behaviorally active and showed a relatively constant rotarod score (Figures 1B,B′) and grip strength (Figures 1C,C′) during the experimental period, both of which scores were significantly worse in the vehicle AR2 mice from 8 weeks. The group receiving 45 mg of WSJP had a significantly better grip strength than the vehicle group from 4 weeks (Figure 1C′). There were no significant differences among the three WSJP treatment groups. All of the mice tolerated the administration of WSJP prescription for 12 weeks.

### Wen-Shen-Jian-Pi Prescription Ameliorates Damage to Spinal Cord Cells in Amyotrophic Lateral Sclerosis Model Mice

**FIGURE 2:**
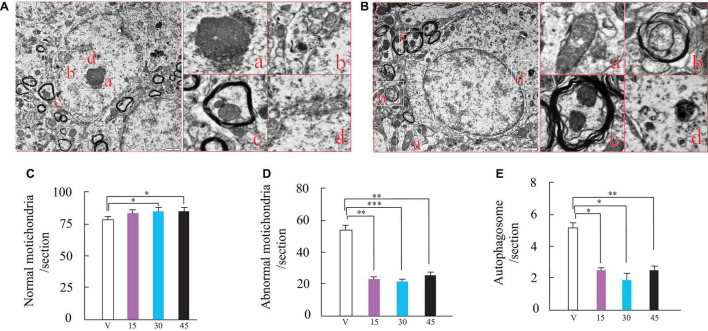
Wen-Shen-Jian-Pi (WSJP) prescription administration for 12 weeks increased normalized mitochondria and decreased abnormal mitochondria and autophagosomes in the AR2 mice. Panels **(A,B)** show transmission electron microscopy images of spinal cord cells from AR2 mice. **(A,a)** Nucleus; **(A,b)** normal mitochondria; **(A,c)** mitochondrial autophagy; **(A,d)** abnormal mitochondria; **(B,a,b)** abnormal mitochondrial autophagy, **(B,c)** normal mitochondrial autophagy and **(B,d)** abnormal mitochondria. The scale bar indicates 1 μm. Frequency histogram of the number of normal mitochondria **(C)**, abnormal mitochondria **(D)** and autophagosomes **(E)** for WSJP-treated AR2 mice [*n* = 6 for each of panels **(C–E)**] and vehicle (water)-treated AR2 mice (*n* = 6); *p < 0.05, **p < 0.001, and ***p < 0.0001 vs. the vehicle-treated mice.

Over time, the number of normal mitochondria, abnormal mitochondria, and autophagosomes in injured spinal cord cells changed gradually (Figures 2A,B). In the injured spinal cord cells, normal mitochondria (usually has a short rod-shaped or elliptical structure, with clear inner ridge structure, smooth outer membrane, and high-density electron clouds between the inner ridges) numbers decreased significantly, whereas abnormal mitochondria (The volume was significantly increased and swollen, the shape was irregular, the shape of the inner ridge was fuzzy and lost, there was a fold, the outer membrane was rough and partially damaged) levels increased significantly (Figures 2A,B). Histological evaluation of spinal cord cells showed significantly higher numbers of normal mitochondria in the WSJP-treated AR2 mice (15, 30, and 45 mg) compared with those in the vehicle group (Figure 2C). At the same time, in the injured cells, the numbers of abnormal mitochondria and autophagosomes (Abnormal autophagosomes showed incomplete outer membrane and leakage) decreased markedly in the WSJP-treated groups compared with those in the cells from the vehicle group (Figures 2D,E). No significant differences were found for the changes in the numbers of normal mitochondria, abnormal mitochondria, and autophagosomes among the WSJP treatment groups.

## Discussion

The results of the present study revealed that the WSJP prescription administered orally to AR2 mice for 12 weeks prevented ALS progression significantly, and ameliorated mitochondria and autophagosome pathology-associated motor neuron cell death. The successful rescue of the ALS phenotype of the AR2 mice, which are a mechanistic model of sporadic ALS, suggested that the WSJP prescription could be used to treat ALS.

We found WSJP-treated AR2 mice gradually gained body weight (Figures 1A,A′). WSJP could significantly reduce ALS-associated weight loss, improve the activity of mouse limbs, the activity persistence of muscles, and the physical flexibility and muscle strength of the mice. The result suggested that oral WSJP treatment might have the efficacy to prevent the development of atrophy in ALS, which is consistent with clinical studies of ALS using other TCM materials (Petrov et al., 2017; Qu et al., 2020). From 8 to 12 weeks, WSJP-treated AR2 mice spent a longer time turning on the pole in rotarod test and had more powerful grip strength than the mice in the vehicle group, whereas the mice in the vehicle group showed a decreasing trend of motor function (Figures 1B,B′,C,C′). These results indicated that the WSJP prescription might have prevented the loss of motor function and even improved the motor function for AR2 mice compared with that of the vehicle group.

Cell survival and metabolism critically rely on mitochondria, which are especially important in neurons (Shigematsu et al., 2021). In neurons, mitochondria have important functions in apoptosis, phospholipid synthesis, and calcium homeostasis, besides their well-known functions in oxidative phosphorylation-driven ATP production. The metabolic demands of neurons are high; consequently, 20% of the ATP produced by the body is consumed by the brain, despite representing only 2% of the body mass (Smith et al., 2019). Mitochondrial maintenance of energy production and calcium homeostasis are particularly important in the maintenance of neuronal function because of neurons’ high metabolic activity and energy requirements. Many proteins associated with sporadic and familial ALS, including SOD1, TAR DNA-binding protein 43 (TDP-43), FUS RNA binding protein (FUS), C9orf72-SMCR8 complex subunit (C9orf72), and glycine/arginine (GR) dipeptide repeat proteins (DPR) associated with repeat amplification of C9ORF72GGGCC, have been shown to interact with mitochondria (Qu et al., 2020). Decreased ATP production and cellular respiration have been demonstrated clearly in ALS, and after the death of patients with sporadic ALS, decreased electron transport complexes I, II, III, and IV activities were observed in the spinal cord (Song and Pan, 2014; Song et al., 2022). In SOD1*^G93A^*transgenic mice, before the onset of motor symptoms, a reduced mitochondrial respiration rate and impaired ATP synthesis were observed in the spinal cord and brain cord, which persisted throughout the disease process (Sun et al., 2020), accompanied by decreased activities of complex I + III, II + II, I, and IV. As expected, WSJP-treated AR2 mice had an almost normal number of normal mitochondria (Figure 2C) and decreased numbers of abnormal mitochondria and autophagosomes (Figures 2D,E). The abnormal mitochondria and autophagosome were seen almost exclusively in the nuclei of motor neurons in ALS (Wang Y. et al., 2021).

The differentiation syndrome in TCM theory for ALS might be divided into seven syndrome types; however, the syndrome type with the largest proportion is deficiency of the yang of the spleen and kidney, at more than 60% (Grimm and Eckert, 2017; see Supplementary Material 1). The TCM, WSJP prescription, is often used in the clinic to treat ALS, and is clinically proven to be effective (Kwak et al., 2010; Petrov et al., 2017; Jin et al., 2019; Qu et al., 2020). To explore the mechanism by which the WSJP prescription treats ALS, we investigated the molecular mechanism and material basis of WSJP *via* network pharmacology. We used the disease database and the TCM database to acquire the potential targets of the prescription in ALS *via* Kyoto Encyclopedia of Genes and genomes (KEGG) and Gene Ontology (GO) using the DAVID tool, established a protein-protein interaction (PPI) network *via* STRING and a Targets network using Cytoscape. The results showed that WSJP might inhibit neuronal death and neuro inflammation *via* multiple pathways to treat ALS (see Supplementary Material 2).

Although traditional Chinese medicine cannot replace western medicine in the treatment of neuro degenerative diseases such as Parkinson’s disease, Alzheimer’s disease; nonetheless, TCM dialectical treatment has achieved certain clinical effects according to the TCM pathogenesis of these degenerative diseases (Wong et al., 2013; Yamashita et al., 2017; Wang Z. Y. et al., 2021). The WSJP prescription, which can tonify the yang of the spleen and kidney and increase the power and energy of qi circulation in the TCM theory, is based on the traditional formula, Si-Jun-Zi decoction (Yamashita and Kwak, 2014), but with increased *Radix Astragali*, *Radix herba Cistanche deserticola*, and *Herba Epimedii*. The prescription also demonstrated powerful effects for muscle weakness and atrophy in many clinical studies (Yu et al., 2014, 2020; Ye and Dong, 2017; Yamashita and Kwak, 2019).

Tonifying the yang of the kidney and spleen, and strengthening and warming qi can delay the progression of ALS in mice and locally improve motor function. Whether the long-term use of this method can delay the progression of ALS also requires further study. In addition, recent studies have suggested that the early onset of neurodegenerative diseases, including Parkinson’s disease and Alzheimer’s disease, is related to the imbalance of intestinal flora (Zhang et al., 2014, 2021). The adjustment of intestinal flora in TCM is mainly carried out by the spleen and coordinated by the liver. Treating neurodegenerative diseases from the spleen might become a new therapeutic target of TCM. Aizawa et al. (2021) found that perampanel, an AMPA receptor antagonists, restored the size of anterior horn cells to that found in age-matched wide-type mice, which indicated that the physiological functions disturbed by increased Ca^2+^ influx *via* abnormal AMPA receptors had been restored (Zhu et al., 2017). Perampanel was effective in asymptomatic AR2 mice and in AR2 mice during disease progression, as reported in a study using adeno-associated virus-mediated *ADAR2* gene delivery. However, the AR2 mice treated with perampanel did not gain body weight, which was recognized an adverse effect of perampanel in clinical trials. TDP-43-related pathology, in which TDP-43 is mislocalized from the nucleus to abnormal cytoplasmic inclusion bodies in motor neurons, is found in the motor neurons of the majority of patients with ALS. This represents a pathological hallmark of ALS and is considered to be closely involved in the pathogenesis of ALS (Zhu et al., 2017). In the present study, we did not measure the increased number of TDP-43-positive neurons, especially those neurons with nuclear and nucleocytoplasmic patterns, nor the subcellular localization of TDP-43 in the AR2 mouse motor neurons. In addition, we did not confirm whether the WSJP-treated AR2 mice showed amelioration of the increased Ca^2+^ influx in ADAR2-depleted motor neurons.

WSJP prescription, which according to TCM, can tonify the yang of the spleen and kidney and improve the power and circulation of qi, can improve clinical symptoms of ALS, slow disease progression, and reduce the loss of motor activity in AR2 mice, and is thus worthy of clinical application.

## Data Availability Statement

The raw data supporting the conclusions of this article will be made available by the authors, without undue reservation.

## Ethics Statement

The animal study was reviewed and approved by the Committee of Animal Handling of the Shanghai University of Traditional Chinese Medicine.

## Author Contributions

WP and TL contributed to conception and design of the study. WP organized the database and wrote the first draft of the manuscript. MW performed the statistical analysis. XZ, CW, and MW wrote sections of the manuscript. All authors contributed to manuscript revision, read, and approved the submitted version.

## Conflict of Interest

The authors declare that the research was conducted in the absence of any commercial or financial relationships that could be construed as a potential conflict of interest.

## Publisher’s Note

All claims expressed in this article are solely those of the authors and do not necessarily represent those of their affiliated organizations, or those of the publisher, the editors and the reviewers. Any product that may be evaluated in this article, or claim that may be made by its manufacturer, is not guaranteed or endorsed by the publisher.
