# Supplementary material for: Mechanism of the Curative Effect of Wen-Shen-Jian-Pi Prescription in the Treatment of Amyotrophic Lateral Sclerosis
Source: Front Aging Neurosci. 2022 Apr 8;14:873224. doi: 10.3389/fnagi.2022.873224 (PMC9024327; doi:10.3389/fnagi.2022.873224)
Supplement: Supplementary file 1 [file Data_Sheet_1.docx]

| Sup-Table1 TCM syndrome type and distribution of patients with ALS | | |
| --- | --- | --- |
|  | n (M/F)) | proportion（%） |
| Deficiency of spleen and kidney | 298（177/121） | 56.23 |
| Deficiency of liver and kidney | 75（47/28） | 14.15 |
| Deficiency of spleen and stomach | 57（34/23） | 10.75 |
| Qi and Blood deficiency | 41（22/19） | 7.74 |
| Phlegmy-damp block | 21（12/9） | 3.96 |
| Blood stasis block | 25（16/9） | 4.72 |
| Damp-heat of middle Jiao | 13（8/5） | 2.45 |

**Supplementary Materials Data Sheet 1**

Sup-Figure 1


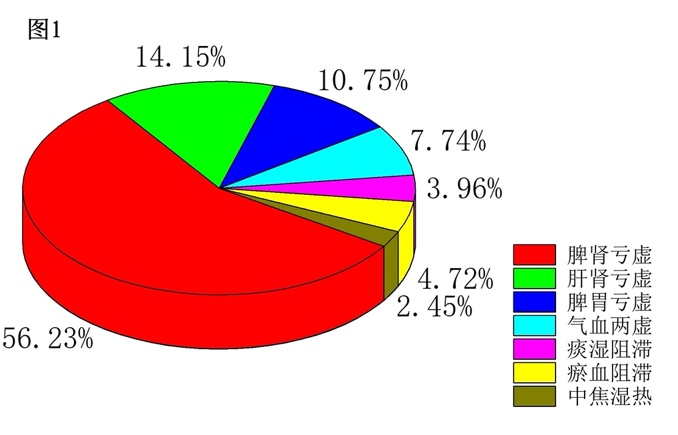


Deficiency of spleen and kidney

Deficiency of liver and kidney

Deficiency of spleen and stomach

Qi and Blood deficiency

Phlegmy-damp block

Blood stasis block

Damp-heat of middle Jiao

| Sup-Table 2 TCM syndrome differentiation of patients with different ALS sub-type [n,(%)] | | | | | | | |
| --- | --- | --- | --- | --- | --- | --- | --- |
|  | Deficiency of spleen and kidney | Deficiency of liver and kidney | Deficiency of spleen and stomach | Qi and Blood deficiency | Phlegmy-damp block | Blood stasis block | Damp-heat of middle Jiao |
| Classic ALS | 78(56.93) | 21(15.33) | 14(10.22) | 15(10.95) | 2(1.46) | 4(2.92) | 3(2.19) |
| Bulbar form ALS | 102(63.75) | 15(9.38) | 17(10.63) | 10(6.25) | 6(3.75) | 9(5.63) | 1(0.63) |
| Flail arm syndrome | 22(44.90) | 7(14.29) | 3(6.12) | 7(14.29) | 4(8.16) | 3(6.12) | 3(6.12) |
| Flail leg syndrome | 47(73.44) | 8(12.50) | 2(3.13) | 1(1.56) | 3(4.69) | 1(1.56) | 2(3.13) |
| Pyramidal tract sign type | 13(30.23) | 15(34.88) | 7(16.28) | 3(6.98) | 2(4.65) | 2(4.65) | 1(2.33) |
| Respiratory ALS | 9(52.94) | 2(11.76) | 1(5.88) | 1(5.88) | 2(11.76) | 2(11.76) | 0(0) |
| Pure lower motor neuron syndrome | 19(48.71) | 5(12.82) | 8(20.51) | 2(5.13) | 1(2.56) | 2(5.13) | 2(5.13) |
| Pure upper motor neuron syndrome | 8(38.10) | 2(9.52) | 5(23.81) | 2(9.52) | 1(4.76) | 2(9.52) | 1(4.76) |
