# Supplementary material for: Mechanism of the Curative Effect of Wen-Shen-Jian-Pi Prescription in the Treatment of Amyotrophic Lateral Sclerosis
Source: Front Aging Neurosci. 2022 Apr 8;14:873224. doi: 10.3389/fnagi.2022.873224 (PMC9024327; doi:10.3389/fnagi.2022.873224)
Supplement: Supplementary file 2 [file Data_Sheet_2.docx]

**Supplementary Materials Data Sheet 2**


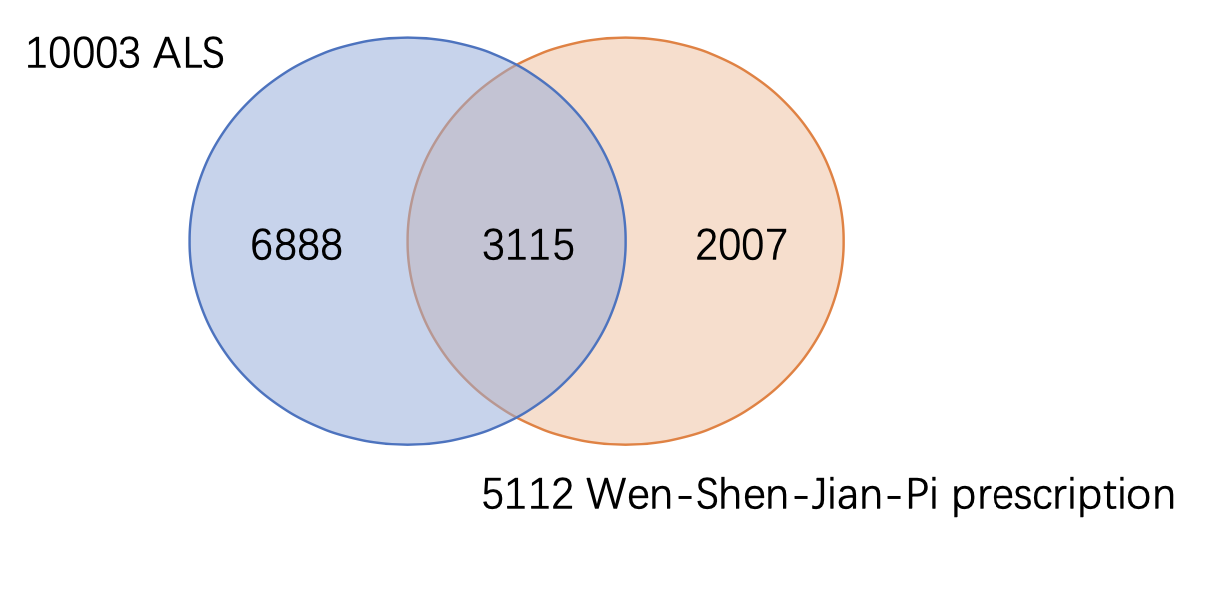


**Sup Figure 1. Potential target map of Wen-Shen-Jian-Pi prescription to treat ALS**


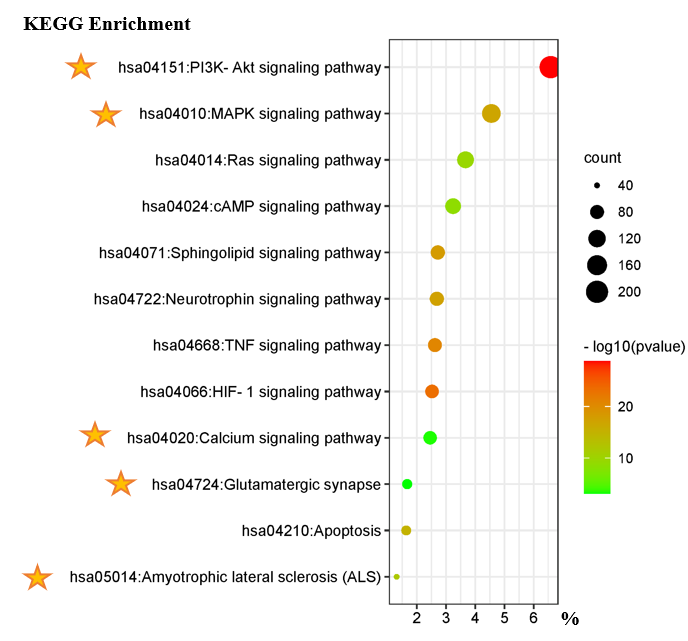


**Sup Figure 2. KEGG pathway enrichment map of Wen-Shen-Jian-Pi prescription to treat ALS**


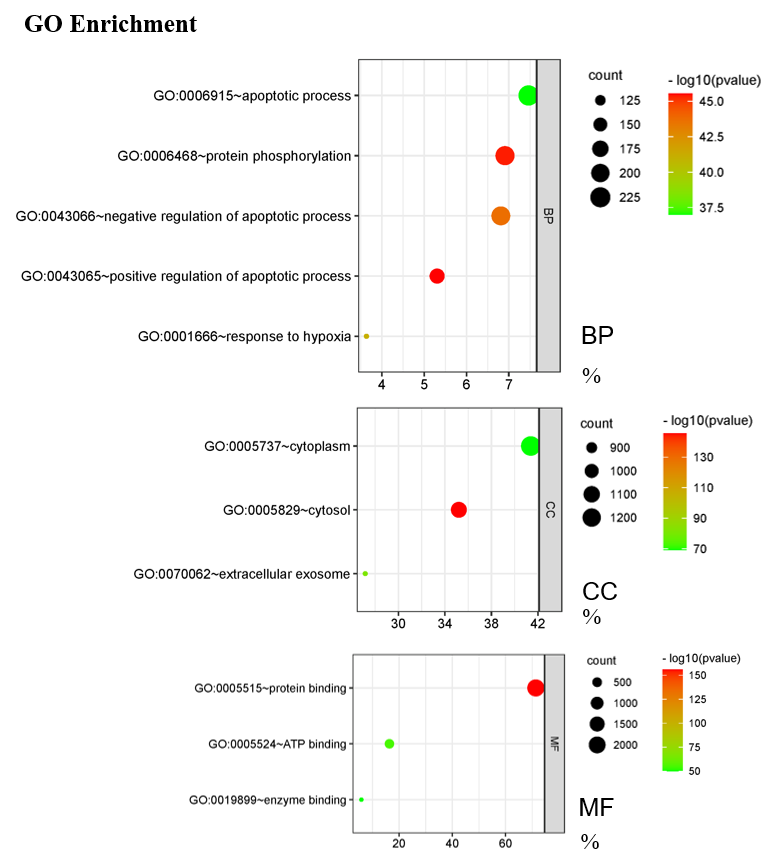


**Sup Figure 3. GO analysis enrichment diagram of Wen-Shen-Jian-Pi prescription to treat ALS**


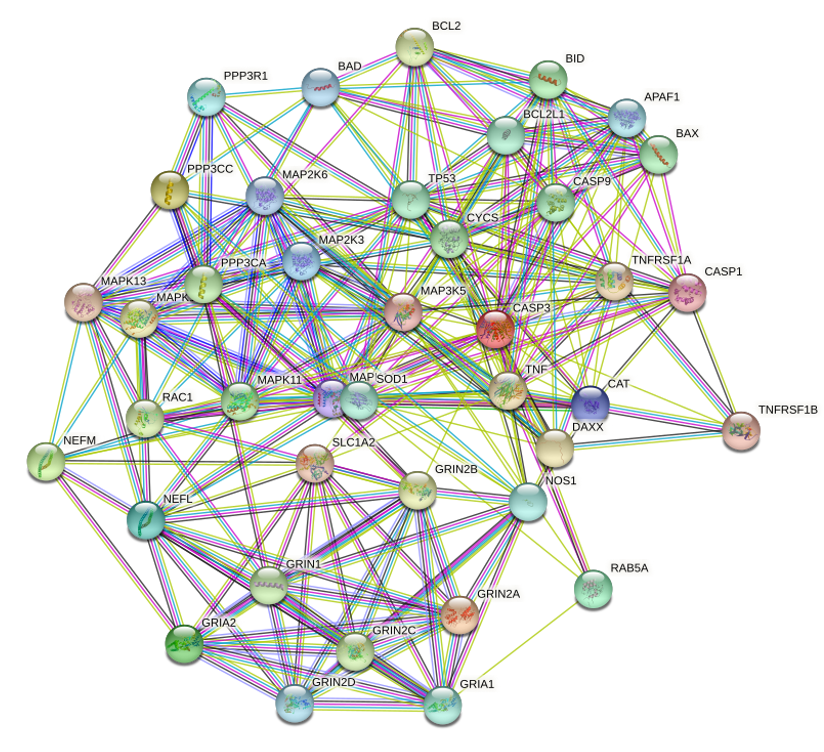


**Sup Figure 4. Protein-protein interaction (PPI) diagram of Wen-Shen-Jian-Pi prescription to treat ALS**


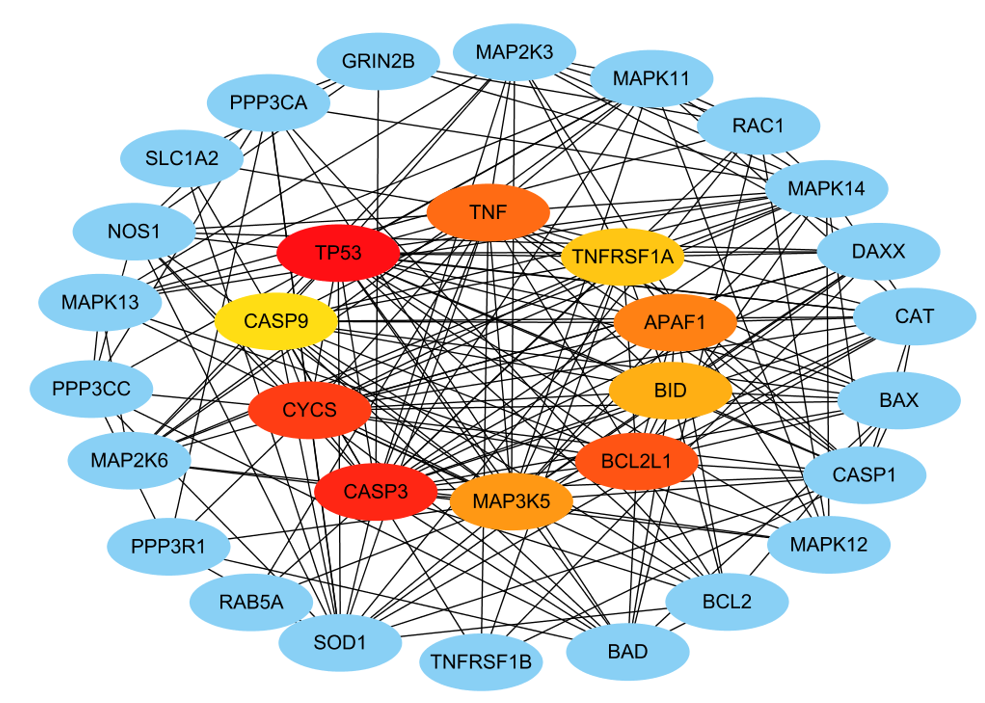


**Sup Figure 5. Target network of Wen-Shen-Jian-Pi prescription to treat ALS**


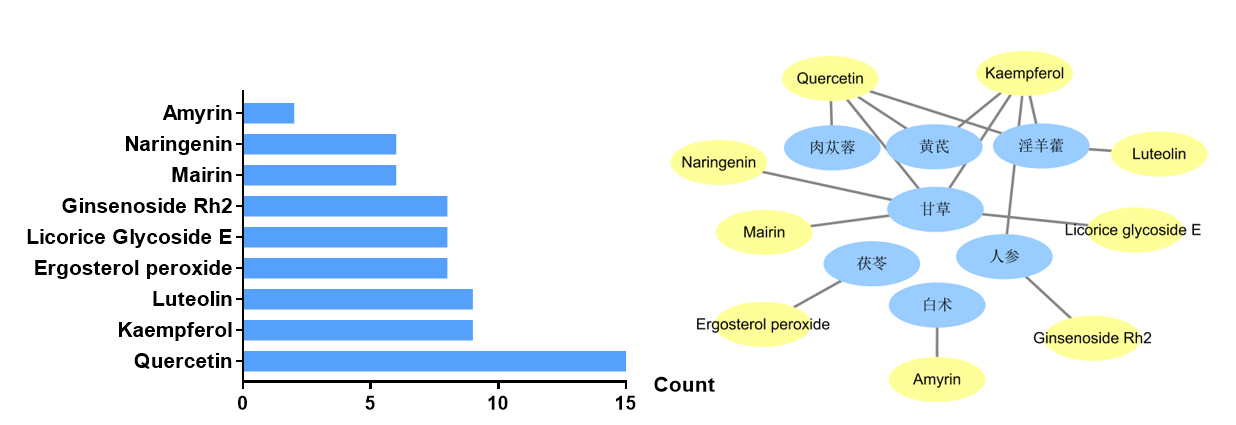


**Sup Figure 6. Potential active ingredients of Wen-Shen-Jian-Pi prescription to treat ALS**

**
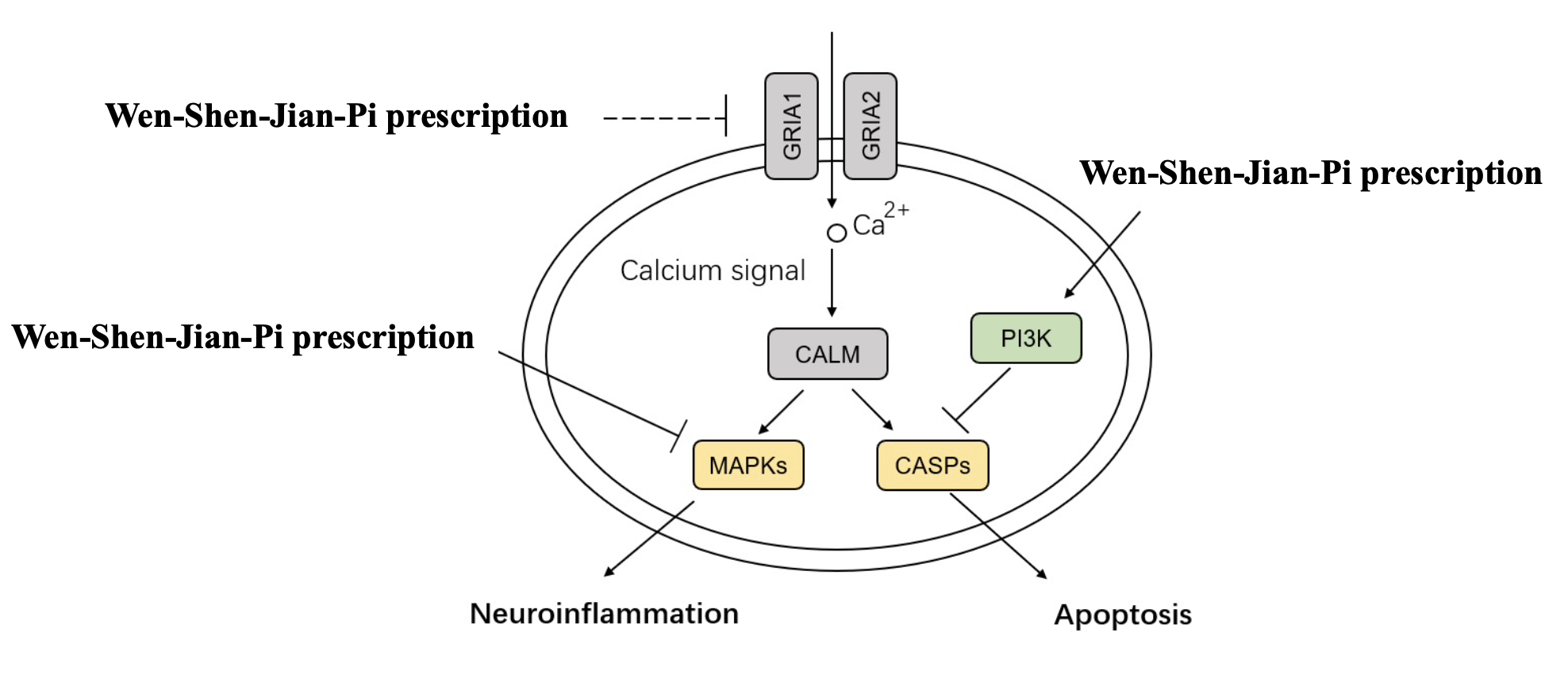
**

**Sup Figure 7. Diagram of the potential mechanism of action of Wen-Shen-Jian-Pi prescription in the treatment of ALS**
